# β-III-spectrin N-terminus is required for high-affinity actin binding and SCA5 neurotoxicity

**DOI:** 10.1038/s41598-022-05762-2

**Published:** 2022-02-02

**Authors:** Sarah A. Denha, Alexandra E. Atang, Thomas S. Hays, Adam W. Avery

**Affiliations:** 1 Department of Chemistry, Oakland University, Rochester, MI USA; 2 Department of Genetics, Cell Biology and Development, University of Minnesota, Minneapolis, MN USA

**Keywords:** Cytoskeletal proteins, Spinocerebellar ataxia

## Abstract

Recent structural studies of β-III-spectrin and related cytoskeletal proteins revealed N-terminal sequences that directly bind actin. These sequences are variable in structure, and immediately precede a conserved actin-binding domain composed of tandem calponin homology domains (CH1 and CH2). Here we investigated in *Drosophila* the significance of the β-spectrin N-terminus, and explored its functional interaction with a CH2-localized L253P mutation that underlies the neurodegenerative disease spinocerebellar ataxia type 5 (SCA5). We report that pan-neuronal expression of an N-terminally truncated β-spectrin fails to rescue lethality resulting from a β-spectrin loss-of-function allele, indicating that the N-terminus is essential to β-spectrin function in vivo. Significantly, N-terminal truncation rescues neurotoxicity and defects in dendritic arborization caused by L253P. In vitro studies show that N-terminal truncation eliminates L253P-induced high-affinity actin binding, providing a mechanistic basis for rescue. These data suggest that N-terminal sequences may be useful therapeutic targets for small molecule modulation of the aberrant actin binding associated with SCA5 β-spectrin and spectrin-related disease proteins.

## Introduction

β-spectrin, in a heterotetrameric complex with α-spectrin, binds actin filaments to form a spectrin-actin cytoskeleton that lines the cytoplasmic surface of the plasma membrane. Recent studies in neurons showed that the spectrin-actin cytoskeleton confers mechanical resiliency to axons^1–3^, regulates axon diameter^4, 5^, organizes microtubule tracks^2, 6^, assembles and maintains axon initial segments and nodes of Ranvier^7–9^, and enables intracellular transport^5, 10^. Moreover, the mammalian β-III-spectrin isoform and the *Drosophila* homolog, β-spectrin, are required for morphogenesis and stability of the large, complex dendritic arbors extended by cerebellar Purkinje cells^11–13^ and dendritic arborization (da) sensory neurons^14^, respectively. Dominant mutations in β-III-spectrin cause the neurodegenerative disease spinocerebellar ataxia type 5 (SCA5)^15^, which is characterized by atrophy of the cerebellar cortex containing Purkinje cell dendrites^16^. A SCA5 missense mutation, L253P, localizes to the β-III-spectrin actin-binding domain (ABD) and induces a ~ 1000-fold increase in actin affinity^17^. In *Drosophila*, this mutation is neurotoxic, and reduces dendrite stability and arbor outgrowth in class IV da neurons^14^. The SCA5 L253P mutation highlights the importance of proper binding of β-spectrin to actin to support neuronal structure and function.

β-spectrin proteins bind actin through an ABD composed of two calponin homology domains (CH1 and CH2) in tandem. The two CH domains are conserved evolutionarily in β-spectrins, and in spectrin-related proteins including α-actinin, filamin and utrophin^18^. Structural studies of numerous tandem-CH ABDs showed that in the absence of actin, CH1 and CH2 are bound to each other in a compact structural state^19–22^. Cryo-EM studies of the β-III-spectrin^23^, filamin A^24^ or utrophin^25^ ABD in complex with actin showed that actin binding is directly mediated by CH1. CH1 engagement with actin requires an ABD conformational “opening” in which CH2 is repositioned to expose an actin-binding site in CH1, and to prevent steric clash between CH2 and actin. The β-III-spectrin SCA5 L253P mutation, which localizes to CH2, shifts the ABD conformational equilibrium towards the open state^23^, explaining the increased actin affinity induced by L253P.

Intriguingly, the recent structural studies of tandem-CH ABDs bound to actin revealed N-terminal sequences, immediately preceding CH1, that also bind actin. Unlike the CH domains, these N-terminal sequences vary greatly in amino acid composition and secondary structure. In the actin-bound state, both the β-III-spectrin and utrophin N-termini take the form of an extended helix^23, 25^, while the filamin A N-terminus forms a helix-loop structure^24^. Truncation of the N-terminus of filamin A^24^ or utrophin^26, 27^ decreases actin binding. Significantly, truncation of the N-terminal sequence preceding the β-III-spectrin CH domains abolishes actin binding^23^. The divergence of N-terminal sequences among spectrin-related proteins suggests the N-termini also confer unique functionality. Indeed, a recent study of the utrophin terminus suggests it directs binding of utrophin to specific actin structures in cells^27^. Further, studies of α-actinin^28^ and plectin^29^ suggest that N-terminal phosphorylation and calmodulin binding regulate actin binding. The in vivo significance of the β-III-spectrin N-terminus, in any species, has yet to be established.

Here, using new *Drosophila* transgenic lines, we examine the functional significance of the β-spectrin N-terminus in vivo. We further explore the functional interaction of the N-terminus with the SCA5, CH2-localized L253P mutation that induces high-affinity actin binding.

## Results

### Delineation of the β-III-spectrin N-terminus

**Figure 1:**
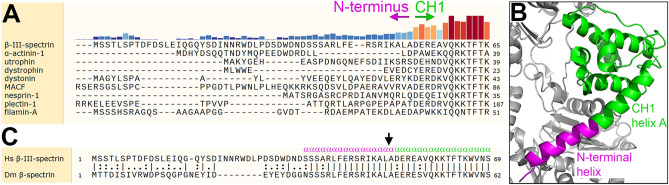
Delineation of N-terminus. (**A**) Alignment of N-terminal amino acid sequences preceding CH1. Tall and dark red bars indicate high conservation, while short and dark blue bars indicate low conservation. Based on increased conservation, we define the first residue of β-III-spectrin CH1 as D53. Amino acids 1–52 constitute the N-terminus. (**B**) Structure of β-III-spectrin CH1 (green) and N-terminal residues N35-A52 (magenta) in complex with actin (grey) (PDB: 6ANU). N-terminal residues S37-A52 form a helix that is continuous with helix A of CH1. (**C**) Alignment of human (Hs) and Drosophila (Dm) β-spectrin showing high conservation in N-terminal residues. Arrow indicates the site of N-terminal truncation chosen for Drosophila ΔN transgenes and binding assays.

Prior cryo-EM analysis showed that the N-terminus of β-III-spectrin forms an extended α-helix that is tightly associated with actin, and is contiguous with helix A of CH1. To clarify the boundary between N-terminus and CH1, the β-III-spectrin sequence was aligned to other tandem-CH ABD proteins to assess amino acid conservation. The alignment shows low conservation of residues in the N-terminal region corresponding to β-III-spectrin residues 1–52, Fig. 1A. In contrast, beginning at β-III-spectrin residue D53, sequence conservation is clearly elevated. Thus, we define D53 as the first residue of the conserved CH1, similar to a prior CH1 demarcation^18^; the novel N-terminal α-helix identified by cryo-EM is composed of N-terminal residues S37–A52, and is contiguous with CH1 helix A that begins at residue D53, Fig. 1B. The N-terminal sequence is well-conserved between human β-III-spectrin and *Drosophila* β-spectrin (Fig. 1C), supporting the functional importance of the N-terminus across species.

### N-terminus is required for β-spectrin function and SCA5 toxicity in *Drosophila*

Our prior biochemical truncation studies showed that N-terminal residues 1–51 are required for binding of the β-III-spectrin to actin in vitro. To evaluate the importance of the N-terminus to β-spectrin function in vivo, new *Drosophila* lines were generated carrying transgenes consisting of an upstream activation sequence (UAS) fused to the *Drosophila* β-spectrin coding sequence, with or without the N-terminal truncation (ΔN; see Fig. 1C). The *UAS-βspec* transgenes were inserted into the *attP154* genomic locus^30^ for equal expression. Rescue experiments were performed to assess the ability of the *βspec* transgenes to support viability. Males carrying *βspec* transgenes were crossed to females carrying an X-chromosome containing the pan-neuronal driver, *elav*-*Gal4,* and an ems-induced mutation (em21) in the endogenous *βspec* gene*. βspec*^*em21*^ is a recessive, loss-of-function allele containing a nonsense mutation in the thirteenth spectrin-repeat domain^31^, resulting in low level expression of a truncated β-spectrin protein, Fig. S1. The *βspec*^*em21*^ allele causes lethality in males and homozygous females. In contrast females heterozygous for *βspec*^*em21*^ are healthy. Pan-neuronal expression of transgenic wild-type β-spectrin (*βspec*^*WT*^) rescued adult viability in ~ 25% of male progeny receiving the *βspec*^*em21*^ mutant X-chromosome, Table 1. In contrast, expression of N-terminally truncated β-spectrin (*βspec*^*∆N-WT*^) failed to rescue *βspec*^*em21*^ lethality. This indicates that the N-terminus is required for β-spectrin function in vivo. In addition, expression of N-terminally truncated β-spectrin (*βspec*^*∆N-WT*^) in heterozygous *βspec*^*em21*^ females reduced viability by ~ 30%. This indicates that the N-terminal truncation mutant is mildly neurotoxic. Moreover, raising *Drosophila* incubation temperature from 22 to 25 °C, to increase expression of the N-terminal truncation mutant, reduced survival by ~ 95%, Table S1 This indicates that toxicity of the N-terminal truncation mutant is dose responsive, consistent with a dominant negative mechanism. Additionally, pan-neuronal expression of *βspec*^*∆N-WT*^ in the absence of *βspec*^*em21*^also caused lethality (Table S2), indicating that *βspec*^*∆N-WT*^ neurotoxicity is not due to a possible functional interaction with the *βspec*^*em21*^ protein fragment.

**Table 1:** Rescue of lethal *em21* mutation by *UAS-βspec* transgenes at 22 °C.

Transgene	Progeny class: total number of adults			
	*em21, elav-Gal4/Y; transgene/* +	*FM6/Y; transgene/* +	*em21, elav-Gal4/* + *; transgene/* +	*FM6/* + *; transgene/* +
*attP154*	0	60	97	68
*UAS-βspec*^*WT*^	42	163	161	162
*UAS-βspec*^*ΔN-WT*^	0	147	112	162
*UAS-βspec*^*SCA5*^	0	122	0	163
*UAS-βspec*^*ΔN-SCA5*^	0	130	133	148

Males homozygous for *UAS-βspec* transgenes or the 3rd chromosome *attP154* landing site, were crossed to females carrying the *βspec*^*em21*^*, elav-Gal4* recombinant chromosome balanced over the *FM6* chromosome.

Using the rescue assay, we further tested how the N-terminus functionally interacts with the CH2-localized L253P mutation. In agreement with our prior results^14^, pan-neuronal expression of β-spectrin carrying the SCA5 missense mutation (L246P in *Drosophila* β-spectrin; *βspec*^*SCA5*^) failed to rescue lethality of *βspec*^*em21*^ males, and dominantly induced lethality in females heterozygous for *βspec*^*em21*^, Table 1. In contrast, the SCA5 mutant with N-terminal truncation (*βspec*^*ΔN-SCA5*^) did not induce lethality in female *βspec*^*em21*^ heterozygotes (Table 1), even when the temperature was elevated to increase transgene expression, Table S1. This indicates that the N-terminus is required for L253P-induced neurotoxicity. Further, the N-terminally truncated SCA5 mutant did not rescue lethality in *βspec*^*em21*^ males at 22 °C or 25 °C. Altogether, these data suggest that the N-terminally truncated SCA5 mutant is neither toxic nor functional.

### N-terminal truncation alleviates SCA5-induced dendritic arbor defects

**Figure 2:**
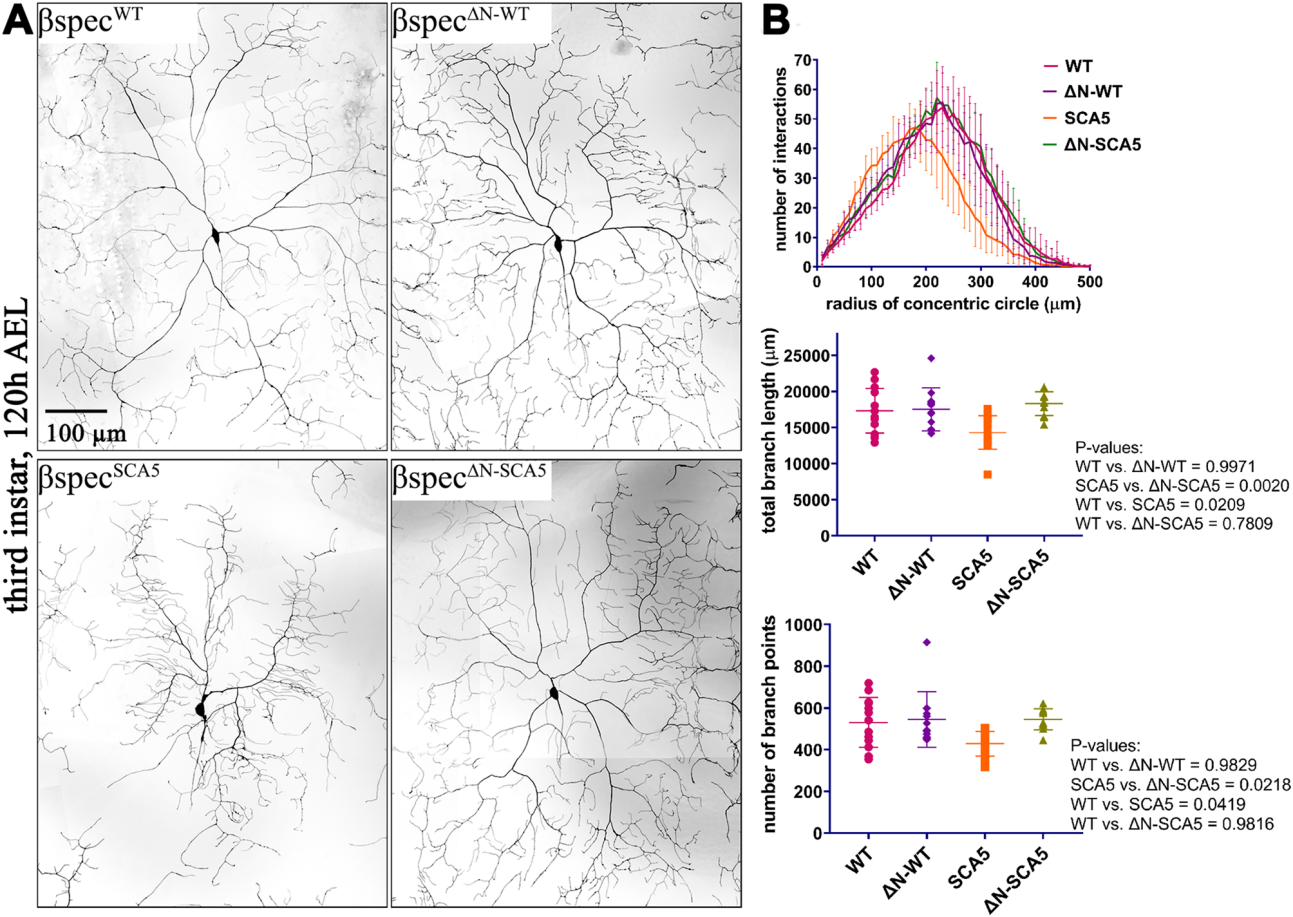
N-terminal truncation alleviates SCA5-induced arborization defects. (**A**) Dendritic arbors of class IV da neurons expressing wild-type Drosophila β-spectrin (*βspec*^*WT*^), N-terminally truncated β-spectrin (*βspec*^*ΔN-WT*^), SCA5 (L246P) β-spectrin with intact N-terminus (*βspec*^*SCA5*^), and N-terminally truncated SCA5 β-spectrin (*βspec*^*ΔN-SCA5*^). Dendritic arbors were documented by fluorescence confocal microcopy in live third instar larvae, 120 h after egg laying (AEL). (**B**) Top, Sholl analyses showing that *βspec*^*SCA5*^, but not *βspec*^*ΔN-WT*^ nor *βspec*^*ΔN-SCA5*^, alter the position of dendritic branches relative to the soma. Middle, quantitation of total branch length showing that only *βspec*^*SCA5*^ significantly alters total branch length relative to *βspec*^*WT*^. Bottom, quantitation showing that only *βspec*^*SCA5*^ impacts total branch points. n = 11–14 neurons for each genotype. Statistical significance was determined by One-way ANOVA followed by multiple comparisons post hoc test. *P*-values are reported to the right of panels.

To determine how N-terminal truncation impacts SCA5-induced dendritic arborization defects, *βspec* transgenes were expressed using the *477-Gal4* driver in *Drosophila* class IV da neurons. Relative to neurons expressing *βspec*^*WT*^, neurons expressing *βspec*^*SCA5*^ showed reduced arborization characterized by a loss of distal dendrites in Sholl analysis, and decreased total branch length and number of branch points (Fig. 2), consistent with our prior characterization^14^. In contrast, neurons expressing *βspec*^*ΔN-SCA5*^ displayed a dendritic arbor morphology indistinguishable from control neurons expressing the wild-type transgene. This indicates that the N-terminus is required for the SCA5 mutation to induce dendritic arbor defects. Moreover, the N-terminally truncated wild-type β-spectrin (*βspec*^*ΔN-WT*^) did not reduce arborization, consistent with mild neurotoxicity observed in rescue assays at 22 °C.

### N-terminus modulates β-spectrin protein level in neurons

**Figure 3:**
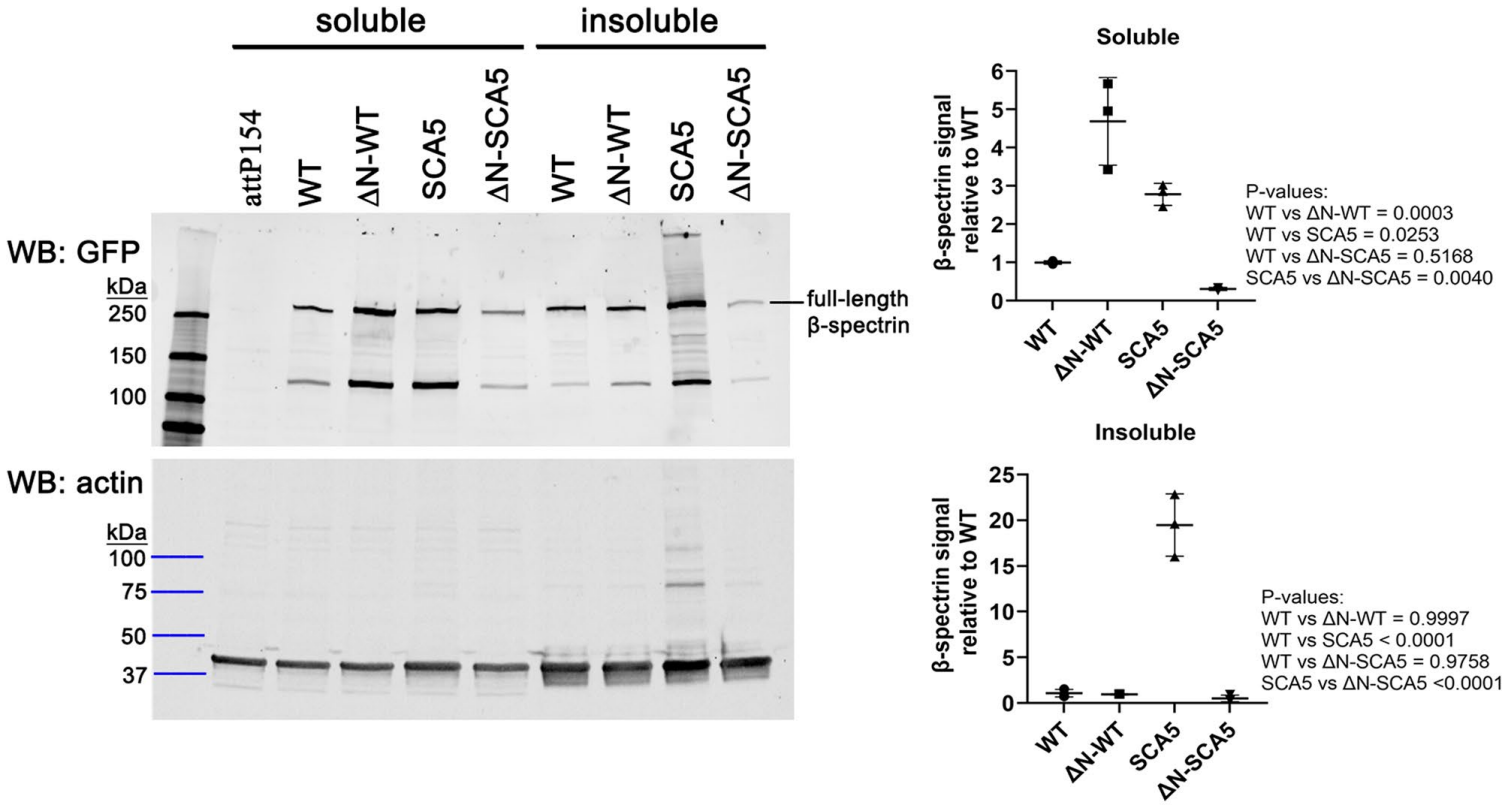
N-terminal truncation impacts β-spectrin protein level. *Left*, western blot of head extracts from adult *Drosophila* expressing *UAS-βspec*^*GFP*^ transgenes under the pan-neuronal driver *elav-Gal4*. As a control, head extracts were also prepared from adult *Drosophila* containing the *attP154* landing site, into which all *UAS-βspec*^*GFP*^ transgenes were inserted. β-spectrin proteins were identified by probing with a GFP antibody. Total actin was probed as a loading control. The GFP and actin western blot images were cropped to show the regions of the blot containing GFP and actin proteins. The full-size gel images are provided in Figs. S2 and S3. *Right*, quantitation of β-spectrin-GFP protein levels from western blot. Head extracts were prepared a total of three times for each genotype. Statistical analysis was performed by One-way ANOVA followed by multiple comparisons post hoc test. *P*-values are reported to the right of panels.

To evaluate the potential impact of N-terminal truncation on β-spectrin protein level, an additional set of *Drosophila* lines were generated containing UAS-transgenes, consisting of β-spectrin, with or without N-terminus, fused at the C-terminus to GFP. The transgenes encoding the β-spectrin-GFP fusion proteins were inserted into the *attP154* genomic locus, as performed for the untagged β-spectrins. In rescue assays, the β-spectrin-GFP fusions performed similar to their untagged counterparts (Table S3), indicating that the GFP tag does not interfere with function. To assess relative abundance of wild-type and N-terminally truncated β-spectrins, female *βspec*^*em21*^ heterozygotes expressing *UAS*-*βspec*^*GFP*^ transgenes under the pan-neuronal *elav-Gal4* driver were collected from rescue assays at 18 °C and head extracts prepared. Using a GFP antibody, western blot analysis of *UAS*-*βspec*^*WT-GFP*^ extracts revealed a prominent band running over 250 kDa, consistent with the predicted size (293 kDa) of the full-length β-spectrin-GFP protein, Fig. 3. Significantly, in the soluble fraction, N-terminally truncated wild-type β-spectrin was ~ fivefold more abundant than wild-type β-spectrin with intact N-terminus. This suggests that the inability of the N-terminally truncated β-spectrin to support viability is not due to insufficient protein level. Instead, it suggests that the N-terminus is essential for β-spectrin function in neurons, presumably to enable actin binding. Moreover, the increased steady-state β-spectrin protein level potentially reflects the effect of N-terminal truncation to stabilize wild-type β-spectrin protein, as observed in thermal denaturation studies^23^.

We likewise evaluated the abundance of SCA5 β-spectrin protein, with and without N-terminus. At 18 °C, a fraction of female *βspec*^*em21*^ heterozygotes expressing the toxic SCA5 mutant β-spectrin survive to adulthood, enabling evaluation of SCA5 mutant protein abundance in head extracts. For SCA5 β-spectrin with intact N-terminus, western blot analysis revealed that soluble SCA5 β-spectrin protein is ~ threefold more abundant than wild-type β-spectrin with intact N-terminus, Fig. 3. SCA5 β-spectrin was also more abundant than wild-type in the insoluble fraction. The increased SCA5 β-spectrin in the insoluble fraction may reflect its high affinity for actin, as the insoluble fraction is enriched in actin. Altogether, the data indicate that the SCA5 mutation, in addition to causing high-affinity actin binding, also causes the mutant β-spectrin protein to accumulate in neurons. It is possible that a high-affinity complex formed between SCA5 β-spectrin and F-actin is resistant to protein turnover. Interestingly, western blotting revealed a fraction of actin with reduced gel mobility in SCA5 β-spectrin head extracts, Fig. 3. Potentially high-affinity binding of SCA5 β-spectrin to F-actin results in post-translational or structural modifications to actin.

Further, the average level of soluble, N-terminally truncated SCA5 β-spectrin protein was 67% lower than wild-type β-spectrin with intact N-terminus, and 89% lower than SCA5 β-spectrin, Fig. 3. In addition, the N-terminally truncated SCA5 β-spectrin did not accumulate in the insoluble fraction, nor cause reduced gel mobility of actin, as observed for SCA5 β-spectrin with intact N-terminus. The lower abundance of the N-terminally truncated SCA5 β-spectrin likely contributes to its the lack of toxicity. However, toxicity was not observed for N-terminally truncated SCA5 β-spectrin when incubation temperature was elevated to increase transgene expression, Table S1. This suggests that the lack of toxicity of N-terminally truncated SCA5 β-spectrin is not solely due to reduced protein level. Instead N-terminal truncation may cause loss of a toxic functional property conferred by the SCA5 mutation.

We evaluated whether the SCA5 mutation or N-terminal truncation impacts the binding of β-spectrin to α-spectrin. Co-immunoprecipitation assays showed that neither the SCA5 mutation nor N-terminal truncation disrupts the co-association of the mutant β-spectrin proteins with α-spectrin in head extracts, Figs. S4 and S5. This indicates that the mutant β-spectrin proteins are not impaired in their ability to bind α-spectrin nor assemble into a spectrin heterotetramer.

### L253P causes protein destabilization independent of the N-terminus

**Figure 4:**
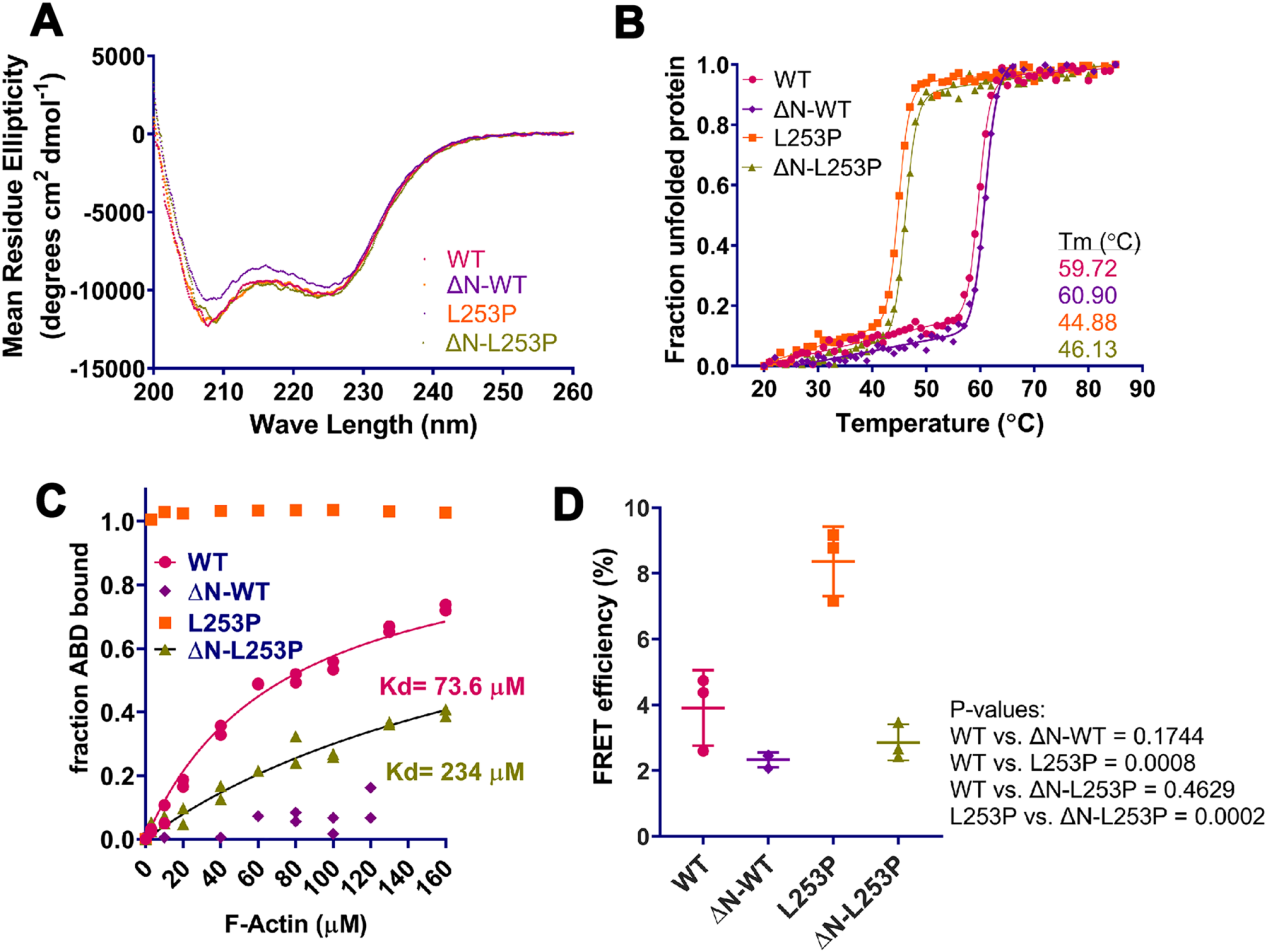
Structural and functional characterization of N-terminally truncated ABDs. (**A**) Circular dichroism (CD) absorption spectra for wild-type and L253P β-III-spectrin ABDs, with or without N-terminus. All ABDs show pronounced α-helical absorption profiles characterized by minima at 208 and 222 nm. (**B**) CD thermal denaturation curves generated by monitoring absorption at 222 nm. All ABDs unfolded with a cooperative, two-state transition, indicating well-folded proteins. Introduction of L253P decreases the Tm’s by ~ 15 °C. In contrast, N-terminal truncation increases the Tm’s by ~ 1 °C. (**C**) In vitro co-sedimentation assays showing that N-terminally truncated human β-III-spectrin ABD with L253P mutation (ΔN-L253P) binds actin with Kd of 234 µM (95% confidence interval: 215.5–253.8 µM), compared to the L253P ABD which is entirely bound at all actin concentrations, or to the wild-type ABD with intact N-terminus (WT) that binds actin with a Kd of 73.6 µM (95% confidence interval: 68.0–79.3 µM). Duplicate data points are plotted for each actin concentration. (**D**) FRET assays monitoring the binding of ABDs to F-actin in live HEK293 cells. L253P increases FRET relative to wild-type. N-terminal truncation blocks L253P-induced actin binding. n = 3. Statistical significance determined by One-way ANOVA followed by multiple comparison post hoc test. P-values are reported to the right of the panel.

To assess how N-terminal truncation impacts the folded state and stability of the SCA5 mutant β-spectrin, circular dichroism spectroscopy was performed. As previously reported, the β-III-spectrin L253P ABD with intact N-terminus showed a pronounced α-helical absorption profile consistent with a well-folded ABD, Fig. 4A. Further, thermal denaturation studies showed that the L253P ABD unfolded in a cooperative, two-state transition with a melting temperature (Tm) of 44.9 °C, Fig. 4B. This Tm is 14.8 °C lower than the Tm of the wild-type ABD with intact N-terminus (59.7 °C). The circular dichroism absorption spectra of the N-terminally truncated L253P ABD similarly showed an α-helical absorption profile indicating a well-folded protein, Fig. 4A. Significantly, the N-terminally truncated L253P ABD unfolded with a Tm of 46.1 °C. Thus, the N-terminally truncated L253P ABD, like the L253P ABD with intact N-terminus, is highly destabilized relative to wild-type. Notably, N-terminal truncation of the L253P ABD or wild-type ABD raised the Tm by ~ 1 °C relative to the L253P or wild-type ABD with intact N-terminus, respectively. We previously reported a 1 °C increase in Tm for the N-terminally truncated wild-type ABD^23^, and suggested that this reflects the loss of an N-terminus that is structurally disordered when not bound to actin.

### N-terminus is required for L253P-induced high-affinity actin binding

To determine whether N-terminal truncation impacts L253P-induced high-affinity actin binding, in vitro co-sedimentation assays were performed. Consistent with high-affinity binding previously reported^17^, the L253P ABD with intact N-terminus was entirely bound to F-actin at all actin concentrations tested (3–140 µM), Figs. 4C, S6 and S7. In contrast, the N-terminally truncated L253P ABD bound actin with very low affinity (Kd = 234 µM). Indeed, the actin-binding affinity of the N-terminally truncated L253P ABD is ~ 3000-fold lower than the affinity of the L253P ABD with intact N-terminus (Kd = 75 nM;^17^, and threefold lower than the affinity of the wild-type ABD with intact N-terminus (74 µM). The binding affinity of the N-terminally truncated L253P ABD is higher than that of the N-terminally truncated wild-type ABD, which did not bind actin with measurable affinity.

To confirm the in vitro co-sedimentation data, we employed a recently developed Förster resonance electron transfer (FRET) assay that detects the binding of the β-III-spectrin ABD to F-actin in live cells^32^. In this assay, HEK293T cells are transfected with a DNA construct consisting of the ABD fused to GFP, and a second construct consisting of the actin-binding peptide Lifeact fused to mCherry. FRET between GFP and mCherry results from the formation of a ternary complex consisting of the ABD-GFP and Lifeact-mCherry bound to an actin filament. Co-expression of the wild-type ABD and Lifeact-mCherry resulted in an average FRET efficiency of 3.9%, Fig. 4D. In contrast, the L253P mutant ABD showed an increased FRET efficiency of 8.4%, indicating that the L253P mutation causes increased actin binding in live cells. Significantly, N-terminal truncation of the wild-type ABD or L253P ABD lowered the FRET efficiency to 2.1% and 2.4%, respectively. Altogether, the biochemical and live cell FRET binding assays demonstrate that the N-terminus is required for the L253P mutation to induce high-affinity actin binding.

## Discussion

The binding of β-spectrin to actin is required for the formation of the plasma membrane-associated spectrin cytoskeleton that is implicated in numerous neuronal functions. Here we investigated the in vivo functional significance of the β-III-spectrin N-terminus, which flanks the conserved tandem-CH ABD, and was recently discovered by cryo-EM, to bind actin. Using *Drosophila*, we demonstrated that the N-terminus is essential for β-spectrin function in neurons. Moreover, we showed that the N-terminus is required for neurotoxicity and dendritic arborization defects induced by L253P. We further demonstrated that the N-terminus is required for L253P-induced high-affinity actin binding, which provides a mechanistic explanation for how N-terminal truncation rescues SCA5 neurotoxicity.

The requirement of the N-terminus for L253P-induced high-affinity actin binding emphasizes the critical role of the N-terminus in the actin-binding mechanism. The N-terminus supports β-spectrin actin binding, at least in part, through its direct interaction with actin. The N-terminus is also destabilizing, Fig. 4. Structural instability conferred by the N-terminus may promote the conformational opening of the CH1-CH2 interface that is required for CH1 to bind actin. Potentially the N-terminus supports actin-binding by additional mechanisms. The N-terminal helix is continuous with CH1 helix A (Fig. 1), which makes contacts with CH2 residues, including L253. Possibly binding of the N-terminus to actin alters CH1-CH2 contacts to facilitate opening of the CH1-CH2 interface. In addition, the N-terminus may be a site of regulation for β-spectrin actin-binding activity. Several amino acids in the β-spectrin N-terminus have been reported to be phosphorylated^33, 34^. Phosphorylation of the α-actinin N-terminus reduces actin binding^28^. Potentially β-spectrin phosphorylation modulates the function of the N-terminus to promote actin binding.

Here we showed that the SCA5 L253P mutation, in addition to causing high-affinity actin binding, also causes the mutant β-spectrin protein to accumulate in neurons, Fig. 3. It is possible that this accumulation also contributes to SCA5 neurotoxicity. In contrast, the N-terminally truncated SCA5 mutant did not accumulate, and instead showed decreased abundance relative to wild-type. N-terminal truncation, by itself, is not destabilizing. Rather, we showed that N-terminal truncation increases protein stability (Fig. 4), and that the N-terminally truncated wild-type β-spectrin was more abundant than wild-type β-spectrin with intact N-terminus, Fig. 3. On the other hand, the L253P mutation is strongly destabilizing, in the presence or absence of the N-terminus, Fig. 4. L253P destabilization may reflect solvent exposure of hydrophobic residues at the CH1-CH2 interface. For example, an X-ray crystal structure of filamin A showed that CH1 residue W142 is buried at the CH interface in the “closed”, actin-unbound state^35^. Cryo-EM of the filamin ABD-actin complex showed that in the “open” conformation, W142 makes hydrophobic contacts with actin^24^, avoiding solvent exposure. Actin binding may stabilize the L253P ABD by burying hydrophobic residues that would otherwise be solvent exposed. In contrast, the N-terminally truncated L253P ABD has very low affinity for actin, and thus remains destabilized and prone to misfolding and protein turnover.

A currently pursued therapeutic strategy for SCA5 is to identify small molecules that bind the L253P β-III-spectrin ABD and reduce its binding to actin^32^. The critical role of the N-terminus in actin binding suggests that small molecules that target the N-terminus may effectively reduce actin binding, similar to N-terminal truncation. In addition, it is possible that the large difference in stability of L253P β-III-spectrin versus wild-type may be leveraged to selectively target mutant β-III-spectrin. We suggest that the reduced protein level of N-terminally truncated SCA5 β-spectrin, but not N-terminally truncated wild-type β-spectrin, is due to enhanced protein turnover of a destabilized SCA5 β-spectrin that cannot bind actin. Thus, a small molecule that reduces actin binding may enhance turnover of the destabilized L253P β-III-spectrin, but not the more stable wild-type protein.

## Materials and methods

### Drosophila studies

#### Drosophila stocks

All stocks were maintained on standard food. The following stocks were obtained from the Bloomington Drosophila Stock Center: *477-Gal4/CyO*, *ppk-CD4-tdTom (10a)/TM6, Tb,* and *elav-Gal4 (C155). attP154* transgenic flies were kindly provided by Norbert Perrimon, Harvard Medical School, Boston. The *βspec*^*em21*^ line was obtained from Ronald Dubreuil, University of Illinois at Chicago, Chicago*.* We previously generated *UAS-βspec*^*WT*^ and *UAS-βspec*^*SCA5*^ fly lines^14^.

#### *Generation of UAS-βspec*^∆N^*transgenic fly lines*

The previously generated pUASTattB-β-spectrin wild-type and pUASTattB-β-spectrin SCA5 DNA constructs^14^ were used as templates in PCR to delete the DNA sequence encoding the N-terminal 44 amino acids of Drosophila β-spectrin. For wild-type or SCA5 template, two PCRs were performed using the oligo set, AAAGGTACCGCCAAGTGAAGTTCATCC and CACACTCTCACGCTCCTCGGCCATGGCTGGGGATCTACGGTT, and the oligo set, AACAGATCCCCAGCCATGGCCGAGGAGCGTGAGAGTGTG and AAAGCTAGCTGCTCCAGTTTCTCCTGC. The resulting two PCR products contained overlapping sequences on either side of the intended deletion. To join the two PCR products, a third PCR was performed in which the two PCR products were used as template, together with the oligo AAAGGTACCGCCAAGTGAAGTTCATCC, which contains a KpnI site, and the oligo AAAGCTAGCTGCTCCAGTTTCTCCTGC, which contains a NheI site. The resulting PCR product contained the 5’ region of the wild-type or SCA5 β-spectrin cDNA, with the sequence encoding the N-terminal 44 amino acids deleted. The wild-type or SCA5 PCR product was then inserted KpnI-NheI into an intermediate DNA vector, pAc5.1b-FSPWT, containing the full-length Drosophila β-spectrin cDNA, digested with the same enzymes. The full-length cDNA encoding the N-terminally truncated wild-type or SCA5 β-spectrin was then subcloned from the pAc5.1b intermediate into pUASTattB using KpnI and XbaI enzymes. The final constructs, pUASTattB-β-spectrin ∆N-WT and pUASTattB-β-spectrin ∆N-SCA5 were sequence verified and inserted into the *attP154* landing site using PhiC31 integrase-mediated transgenesis conducted by BestGene, Inc.

#### *Generation of UAS-βspec*^*GFP*^* transgenic fly lines*

A multi-step protocol was followed to fuse mEGFP to the 3′ end of the fly β-spectrin coding sequence. PCR were performed to amplify a 3′ region of the fly β-spectrin coding sequence, using pUASTattB-β-spectrin wild-type as template, together with forward primer, AAACGCCGGCGAGGGTCACGAAGG and the reverse primer, CTCCTCGCCCTTGCTCACCATCTTTTTCTTTAAAGTAAAAAACG. The reverse primer contains sequence complementary to the 3′ end of the β-spectrin coding sequence and sequence complementary to the 5′ end of mEGFP. A second PCR was performed to amplify mEGFP using the forward primer, ATGGTGAGCAAGGGCGAGGAG and the reverse primer, AAATCTAGACTCGTTCTTCTCTTGCTTATGGTTGCGTTACGGCTGTTACTTTACTTGTACAGCTCGTCCATGCC. The reverse primer includes a short β-spectrin 3’UTR sequence, attached to the 3′ end of the mEGFP coding sequence. The mEGFP DNA used as template in the PCR was synthesized by IDT DNA Technologies. A third PCR was performed to fuse the PCR products generated in the first two PCRs. In the third PCR, the PCR products from the first two PCRs were used as template, and combined with the forward primer, AAAGGTACCCGCCGGCGAGGGTCACGAAGG, containing KpnI and MreI restriction sites, and the reverse oligo AAATCTAGACTCGTTCTTCTCTTGCTTATGGTTGCGTTACGGCTGTTACTTTACTTGTACAGCTCGTCCATGCC, containing XbaI site. The resulting PCR product was a fusion of the 3′ coding sequence and mEGFP. The fusion DNA was inserted KpnI-XbaI into pcDNA3.1 digested with the same restriction enzymes, to generate the intermediate construct termed pcDNA3.1-fly-β-spec CT-GFP. The β-spectrin 3′ coding sequence with GFP fusion was subcloned from pcDNA3.1-fly-β-spec CT-GFP into pUASTattB-β-spectrin constructs using MreI and XbaI restriction enzymes. The final pUASTattB-β-spectrin-mEGFP constructs were sequence verified and transgenesis performed using the *attP154* landing site at BestGene, Inc.

#### Rescue experiments

Female flies containing a recombinant X chromosome *βspec*^*em21*^*, elav-Gal4,* balanced over *FM6*, were crossed to male flies homozygous for *UAS-βspec* transgenes, or *attP154* landing site, on the third chromosome. All classes of adult progeny were scored. Fly incubation temperature ranged from 18 to 25 °C, as indicated in the Tables containing rescue assay data.

#### Dendritic arbor analysis

In Fig. 2, female flies carrying *477-Gal4; ppk-CD4-tdTom/TM6* transgene were crossed to *UAS-βspec* males at 22 °C. Eggs were collected from grape juice plates containing yeast paste every 4 h. Third-instar female larvae 120 h after egg laying (AEL) were imaged using HC PL APO 20x/0.80 NA dry objective and and a Leica DMi8 inverted microscope equipped with X-Light V2 spinning-disc (CrestOptics), LDI laser unit (89 North), and Photometrics Prime 95B CMOS camera. Z-stack images of class IV da neurons were obtained from A3 and A4 abdominal segments (1–3 neurons per larva) and processed to generate maximum intensity projections using Metamorph software. Reconstructions of the arbors were generated using Photoshop 21.0.1. Sholl analysis, and total branch length and branch point quantitation were performed as previously described using ImageJ software^14, 36^. Statistical analyses on total branch length and number of branch points were performed in Prism 8 (Graphpad) using unpaired two-tailed t test (n = 10 for *βspec*^*WT*^ and *βspec*^*SCA5*^, n = 11 for *βspec*^*∆N-WT*^ and *βspec*^*∆N-SCA5*^).

#### Western blot analysis

Males homozygous for *UAS-βspec-GFP* transgenes or *attP154* landing site were crossed to females containing *βspec*^*em21*^*, elav-Gal4/FM6.* Flies were incubated on standard food at 18 °C. Adult female progeny containing *βspec*^*em21*^*, elav-Gal4* and *UAS*- *βspec-GFP* transgenes were collected and stored at − 80 °C. To separate fly heads from bodies, a microtube containing flies was frozen in liquid nitrogen and then vigorously shaken. Heads were collected and homogenized using a G-tube with pestle (Bio Plas, Inc) in RIPA buffer (0.05 M HEPES, 0.001 M EDTA, 0.5 M lithium chloride, Complete protease inhibitor cocktail, no EDTA (Roche), 1% IGEPAL CA-630, and 0.7% DOC) at a ratio of 2 µL buffer per head. The supernatants containing soluble proteins were mixed with 4X Laemmli sample buffer (Bio-Rad) after centrifugation at 17,000 RCF and 4 °C for 15 min. The pellets (insoluble fraction) were resuspended in 1X sample buffer. SDS-PAGE was performed on the soluble and insoluble protein samples using 12% SDS-acrylamide gels. Proteins were transferred from gels to Immobilon-FL membranes (Millipore). Membranes were blocked with 1% casein (Hammersten; Alfa Aesar) in 1 × PBS-L (9 g NaCl, 2 g Na_2_HPO_4_, 0.83 g NaH_2_PO_4_·H_2_O, dissolved in 1 L H_2_O) for 1 h at room temperature. Then the membranes were probed overnight at 4 °C with the following primary antibodies diluted in 1 × PBS-L with 1% casein and 0.1% Tween 20: 1:2000 α-actin JLA20 (Developmental Studies Hybridoma Bank), 1:2000 α-GFP (Millipore) or 1:1000 *Drosophila* β-spectrin antibody. Primary antibody solution was removed and membranes washed with 1 × PBS plus 0.1% Tween 20. The membranes were probed for 1 h with the following secondary antibodies: α-mouse IRDye 800CW (LI-COR Biosciences), α-rabbit IRDye 680RD (LI-COR Biosciences), or α-guinea pig IRDye 680RD (LI-COR Biosciences), diluted to 1:5000 in 1xPBS-L with 1% Casein, 0.1% Tween 20, and 0.01% SDS. Secondary antibodies were removed and membrane washed with 1 × PBS plus 0.1% Tween 20. Membranes were imaged on a Sapphire scanner (Azure) using 680 nm and 800 nm channels. Protein band fluorescence intensities were quantified in ImageStudio lite (LI-COR Biosciences) by drawing rectangular boxes of uniform size around each protein band. Local fluorescence background signal was subtracted using the median pixel intensity measured under the upper and lower bounds of each measurement box.

#### Co-immunoprecipitation assay

*Drosophila* head extracts were prepared as described above. After, homogenization and clarification by centrifugation, a Bradford assay was performed to determine lysate protein concentrations. A sample of each lysate (80–100 µg) was collected and mixed with 2 × Laemmli sample buffer. Remaining lysate supernatants were clarified by inversion with Protein A agarose resin for 1 h at 4 °C. Then ~ 500 µg total lysate protein was incubated with either 10 µg anti-*Drosophila* α-spectrin 3A9 (Developmental Studies Hybridoma Bank) or 10 µg normal mouse IgG (Millipore) antibodies for 1 h at 4 °C, with inversion. Protein A agarose beads equilibrated with RIPA buffer was then added to lysate/antibody mixtures and incubated with inversion overnight at 4 °C. Then, Protein A agarose beads were pelleted at 760×*g* for 1 min and washed 3 × with RIPA buffer. The protein samples were eluted by boiling the beads with 1X Laemmli sample buffer for 5 min at 85 °C.

#### Protein expression and purification

The coding sequences for L253P β-III-spectrin ABD with or without N-terminus were PCR amplified from pET-30a-ABD L253P^17^ using the following forward primers for intact N-terminus AAACACCTGCAAAAAGGTATGAGCAGCACGCTGTCACCC or truncated N-terminus AAACACCTGCAAAAAGGTGCAGATGAACGAGAAGCTGTGC, and the reverse primer AAATCTAGACTACTTCATCTTGGAGAAGTAATGGTAGTAAG. PCR products were digested with AarI and XbaI restriction enzymes and ligated into the compatible ends in pE-SUMOpro (LifeSensors) generated by BsaI digestion. The final constructs, pE-SUMO-ABD L253P and pE-SUMO-A52-ABD L253P, together with the previously generated pE-SUMO-ABD WT and pE-SUMO-A52-ABD WT constructs^23^, were transformed into Rosetta 2 (DE3) *E. coli* (Novagen). Bacteria were gown in 1 L of LB broth with ampicillin (100 µg/mL) and chloramphenicol (34 µg/mL) and SUMO-ABD protein expression induced with 0.5 mM IPTG, for 6 h, at 300 rpm and room temperature. Bacteria were pelleted at 4000 rpm and 4 °C for 30 min, and pellets stored at − 20 °C until further use. Protein extraction and purification proceeded as described previously^23^, except that a final gel filtration chromatography step was not performed due high purity of ABD protein. Buffer exchange was performed by dialysis in 10 mM Tris, pH 7.5, 150 mM NaCl, 2 mM, MgCl_2_, and 1 mM DTT using Slide-A-Lyzer, 10,000 MWCO cassettes (Thermo Scientific).

#### Circular dichroism spectroscopy

Purified ABD proteins were diluted to 200 ng/µL in buffer containing 10 mM Tris, pH 7.5, 150 mM NaCl, 2 mM, MgCl_2_, and 1 mM DTT. Circular dichroism absorption spectra and thermal denaturation data were collected in a Jasco J-810 spectropolarimeter with Peltier temperature controller, as described previously^23^. Raw absorption data was converted to mean residue ellipticity using an equation described previously^23^. For thermal denaturation absorption data measured at 222 nm, melting temperatures were calculated in Prism 8 (Graphpad) using nonlinear regression to fit a two-state transition equation previously described^23^.

#### Actin co-sedimentation binding assays

Actin was purified from rabbit muscle acetone powder acquired from Pel-Freez Biologicals. To extract actin, acetone powder was incubated in pre-chilled water (1 g / 20 mL) for 30 min followed by vacuum-filtration using Whatman #4 filter paper. One Complete protease inhibitor cocktail, without EDTA, mini tablet (Roche) was added to the filtrate. Actin was then polymerized to F-actin by addition of 30 mM KCl for 1 h at room temperature. F-actin was pelleted by centrifugation at 80,000 RPM at 4 °C, for 30 min, in a TLA-100.3 rotor (Beckman). Supernatants were decanted and F-actin depolymerized by resuspension and homogenization in G-buffer containing 5 mM Tris, pH 7.5, 0.5 mM ATP, and 0.2 mM CaCl_2_. Homogenized actin was clarified by centrifugation at 70,000 RPM and 4 °C for 10 min in a TLA-100.3 rotor. Actin was polymerized to F-actin by addition of 2 mM MgCl_2_ for 30 min at room temperature. F-actin was pelleted at 80,000 RPM for 30 min at 4 °C in a TLA-100.3 rotor. The F-actin was resuspended and homogenized in F-buffer containing 10 mM Tris, pH 7.5, 150 mM NaCl, 0.5 mM ATP, 2 mM MgCl_2_, 1 mM DTT. Immediately before binding assays, ABD proteins were clarified by centrifugation at 40,000 RPM for 30 min at 4 °C in a TLA-100.3 rotor. ABD and F-actin concentrations were determined by Bradford assay. Binding reactions were performed by combining 2 µM ABD with varying concentrations for F-actin (3–160 µM), and incubating reactions for 30 min at room temperature. To pellet F-actin and actin-bound ABD, binding reactions were centrifuged at 50,000 RPM, for 30 min at 25 °C, in a TLA-100 rotor (Beckman). Supernatants containing unbound ABD protein were sampled and mixed with 1X Laemmli sample buffer. In Fig. S6, pellet samples were also resuspended in 1X Laemmli sample buffer. Supernatant samples were separated by SDS-PAGE and gels stained using Coomassie Brilliant Blue R-250 solution. Destained gels were imaged using the 680 nm channel of an Azure Sapphire imager. ABD band intensities were quantified from fluorescent gel images using Image Studio Lite version 5.2 software. Raw fluorescence intensity data were converted to ABD protein concentration using a standard curve generated from a Coomassie stained gel loaded with varying amounts of ABD protein. Dissociation constants (Kd) were determined in Prism 8 (Graphpad) by nonlinear regression to fit a one site specific binding equation with B max constrained to 1, as described previously^17^.

#### Native-PAGE

ABD and BSA proteins were clarified by centrifugation at 40,000 RPM for 30 min at 4 °C in a TLA-100.3 rotor. ABD and BSA concentrations were determined by Bradford assay. Varying concentrations of ABD proteins (2–8 µM) were prepared in F-buffer containing 10 mM Tris, pH 7.5, 150 mM NaCl, 0.5 mM ATP, 2 mM MgCl_2_, 1 mM DTT. BSA protein was prepared at concentration of 2 µM, also in F-buffer. Samples were mixed with 2X Native Sample Buffer (Bio-Rad). Native-PAGE was performed using a 12% acrylamide gel and ice-cold Tris–glycine buffer (Bio-Rad). Gels were stained using Coomassie Brilliant Blue R-250 solution. The destained gel was imaged using the 785 nm channel of an Azure 600 imager.

#### Live cell FRET binding assays

Mammalian cell DNA constructs were generated to express ABD proteins fused at the C-terminus to mEGFP, to be used as FRET donors. For these constructs, the mEGFP coding sequence was PCR amplified using the forward primer CTTGGTACCACCATGGTGAGC, containing a KpnI site, and the reverse primer AAATCTAGACTACTTGTACAGCTCGTCCATGCC, containing a XbaI site, followed by digestion and ligation into pcDNA3.1, resulting in the intermediate construct pcDNA3.1-mEGFP. For PCR amplification of the ABD coding sequence, the forward primer AAAAAGCTTACCACCATGAGCAGCACGCTGTCACCC, or AAAAAGCTTACCACCATGGCAGATGAACGAGAAGCTGTGC, for full-length or N-terminally truncated ABD, respectively, was used with the reverse primer AAAGGTACCCTTCATCTTGGAGAAGTAATGG. The amplified ABD sequences were digested with HindIII and KpnI, and ligated 5′ of mEGFP in pcDNA3.1-mEGFP. The final FRET donor constructs pcDNA3.1-β-III-spectrin ABD-mEGFP with or without the N-terminal truncation were sequence verified. The pcDNA3.1-Lifeact-mCherry FRET acceptor construct was previously described^32^. HEK293T cells were acquired from the American Tissue Culture Collection (ATTC) and were transiently transfected using Lipofectamine 3000 (ThermoFisher Scientific) with either pcDNA3.1-β-III-spectrin ABD-mEGFP donor construct (D), or co-transfected with pcDNA3.1-β-III-spectrin ABD-mEGFP and pcDNA3.1-Lifeact-mCherry donor and acceptor constructs (DA), in 6-well microplates. Cells were harvested 24 h after transfection by dissociation with TrypLE (Gibco), followed by TrypLE inactivation in 1 × DMEM (4.5 g/L D-glucose and 110 mg/L sodium pyruvate) supplemented with 10% FBS (Gibco) and 1% penicillin/streptomycin (Gibco), and then washing in 1 × DPBS (Gibco). Fluorescence lifetime measurements were performed using time-correlated single photon counting method in a FS5 spectrofluorometer equipped with an EPL-472 pulse laser with 5 MHz frequency (Edinburgh Instruments). mEGFP emission signals were detected at 510 nm until the fluorescence decay reached 1000 photon counts within a 50 ns time window. The lifetime (τ) of mEGFP in D and DA samples was obtained by fitting the fluorescence decay data using Fluoracle software (Edinburgh Instruments) reconvolution analysis with instrument response function correction. The FRET efficiency was calculated using the equation:

$${\text{E}} = 1 - \frac{{{{\tau DA}}}}{{{\tau D}}}$$

#### Sequence alignments

For Fig. 1, amino acid sequences of β-spectrin and related proteins were aligned using Clustal Omega 1.2.4 algorithm in SnapGene 5.1.7.

## Supplementary Information

Supplementary Information.

## Data Availability

Source data generated during and/or analysed during the current study are available from the corresponding author on reasonable request.
