# Supplementary material for: β-III-spectrin N-terminus is required for high-affinity actin binding and SCA5 neurotoxicity
Source: Sci Rep. 2022 Feb 2;12:1726. doi: 10.1038/s41598-022-05762-2 (PMC8810934; doi:10.1038/s41598-022-05762-2)
Supplement: Supplementary file 1 — Supplementary Information. [file 41598_2022_5762_MOESM1_ESM.pdf]

Supplementary Information

β-III-spectrin N-terminus is required for high-affinity actin binding and SCA5 neurotoxicity

Sarah A. Denha[1], Alexandra E. Atang[1], Thomas S. Hays[2], and Adam W. Avery[1,2*]

[1]Department of Chemistry, Oakland University, Rochester, MI

[2]Department of Genetics, Cell Biology and Development, University of Minnesota, Minneapolis, MN

*Corresponding Author: Adam W. Avery
Email: awavery@oakland.edu

Supplementary Table S1

Table S1. Rescue of lethal *em21* mutation by *UAS-βspec* transgenes at 25˚C

transgene	Progeny class: total number of adults			
	em21, elav-Gal4/Y; transgene/+	*FM6/Y; transgene/+*	*em21, elav-Gal4/+; transgene/+*	*FM6/+; transgene/+*
attP154	0	147	172	173
UAS-βspec^{WT}	19	125	145	145
UAS-βspec$^{\Delta N\text{-}WT}$	0	123	9	157
UAS-βspec^{SCA5}	0	88	0	150
UAS-βspec$^{\Delta N\text{-}SCA5}$	0	87	158	138

Males homozygous for *UAS-βspec* transgenes or the 3rd chromosome *attP154* landing site, were crossed to females carrying the *βspec*^{em21}*, elav-Gal4* recombinant chromosome balanced over the *FM6* chromosome.

Supplementary Table S2

Table S2. Neurotoxicity of *UAS-βspec* transgenes at 25°C

transgene	*elavGal4/+;; transgene/+*		
	Number Pupal cases	Number Adults	% Adults/pupal cases
attP154	247	259	104
UAS-βspec^{WT}	298	292	98.0
UAS-ΔN-βspec^{WT}	381	156	40.9
UAS-βspec^{SCA5}	335	16	4.78
UAS-ΔN-βspec^{SCA5}	338	321	95.0

Males homozygous for *UAS-βspec* transgenes or the 3rd chromosome *attP154* landing site, into which *UAS-βspec* transgenes are inserted, were crossed to females homozygous for *elav-Gal4* transgene.

Supplementary Table S3

Table S3. Rescue of lethal *em21* mutation by *UAS-βspec*^{GFP} transgenes

transgene	Progeny class: total number of adults			
	em21, elav-Gal4/Y; transgene/+	*FM6/Y; transgene/+*	*em21, elav-Gal4/+; transgene/+*	*FM6/+; transgene/+*
attP154	0	172	192	156
UAS-βspec$^{WT\text{-}GFP}$	32	206	205	173
UAS-βspec$^{\Delta N\text{-}WT\text{-}GFP}$	0	175	3	173
UAS-βspec$^{SCA5\text{-}GFP}$	0	164	0	179
UAS-βspec$^{\Delta N\text{-}SCA5\text{-}GFP}$	0	130	195	216

Males homozygous for *UAS-βspec-GFP* transgenes, or the 3rd chromosome *attP154* landing site, were crossed to females carrying the *βspec*^{em21}*, elav-Gal4* recombinant chromosome balanced over the *FM6* chromosome.

Supplementary Figure S1

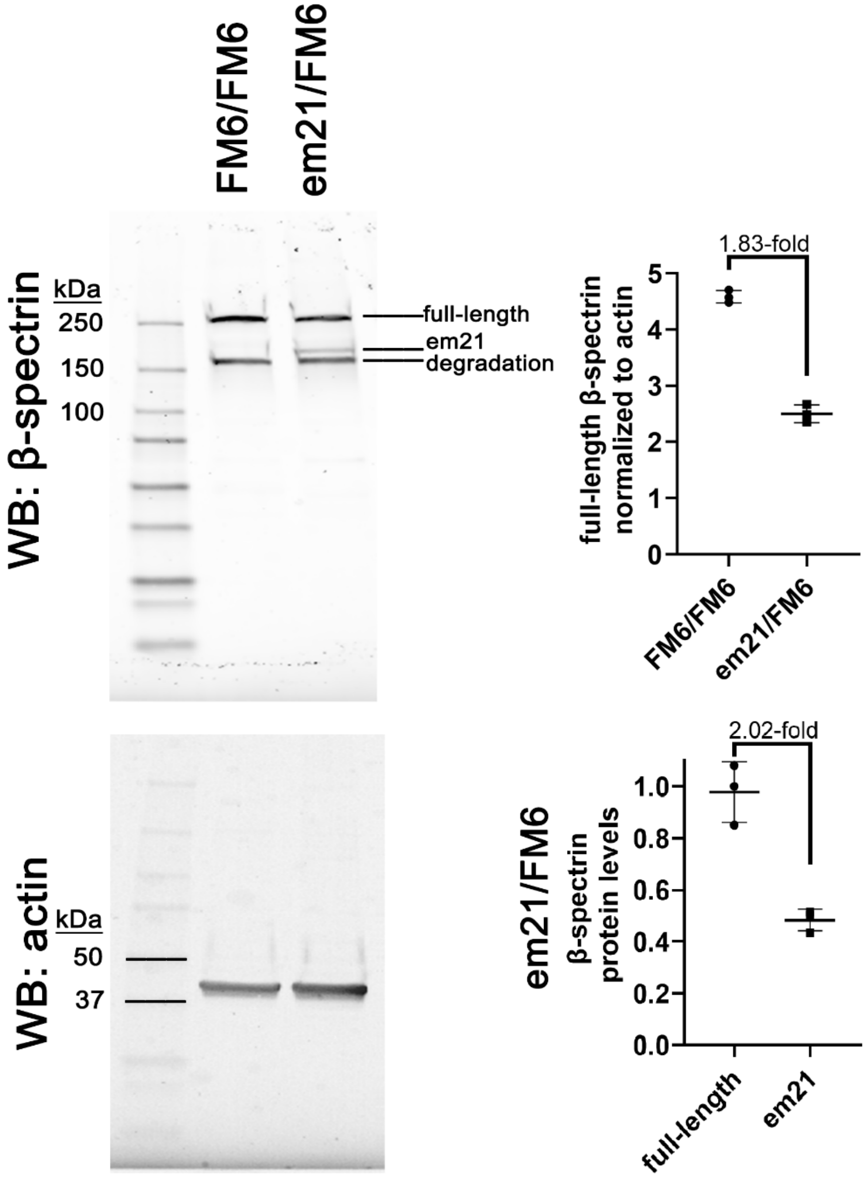

Figure S1. The *βspec*^{em21} allele encodes a truncated protein with reduced expression level. *Drosophila* head extracts were prepared from flies homozygous for the *FM6* X-chromosome balancer containing the endogenous, wild-type *βspec* gene, or flies containing the *FM6* balancer in combination with the *βspec*^{em21} mutant X-chromosome (*FM6*/*βspec*^{em21}). Western blotting of extracts was performed using an antibody specific to *Drosophila* β-spectrin, spectrin-repeat domains 7-11[1]. Western blot of *FM6/FM6* extracts shows a two-band pattern: one band at ~250 kDa, corresponding to full-length β-spectrin, and a second band at ~150 kDa, corresponding to a degradation product. In flies containing the *FM6*/*βspec*^{em21}, an additional band running at ~170 kDa is present. This 170 kDa band corresponds to the truncated em21 protein terminating in spectrin-repeat domain 13[2]. Full-length β-spectrin protein is ~50% lower

in abundance in *FM6*/*βspec*^{em21} extracts relative to *FM6/FM6* extracts, consistent with the 50% reduction in wild-type *βspec* allele number in *FM6/FM6* versus *FM6*/*βspec*^{em21} flies. In *FM6*/*βspec*^{em21} extracts, the full-length β-spectrin protein is ~2-fold more abundant than the truncated em21 protein.

Supplementary Figure S2

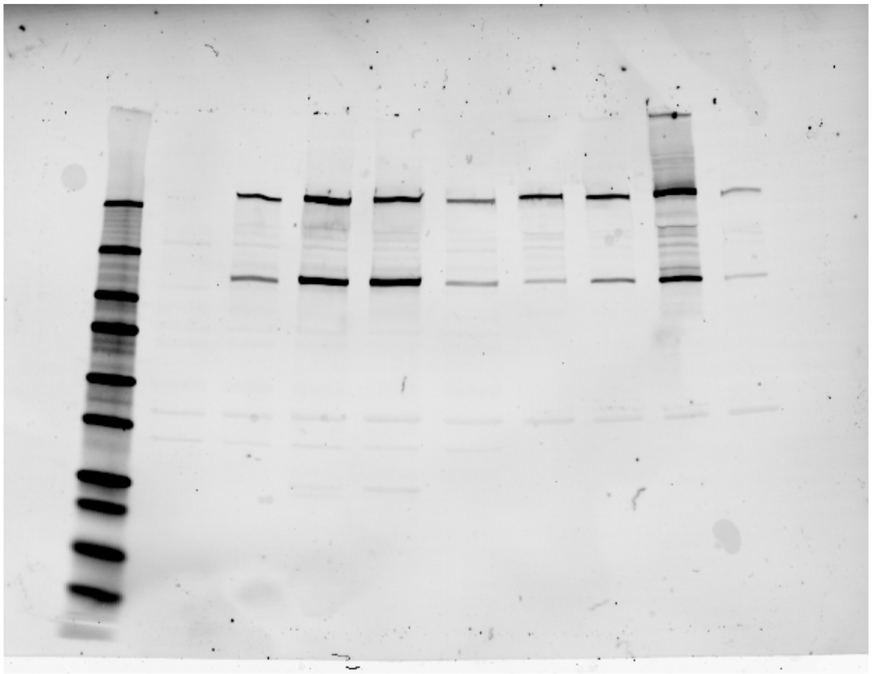

Supplementary Figure S2. Uncropped GFP western blot image. A cropped portion of this GFP western blot image is presented in Figure 3.

Supplementary Figure S3

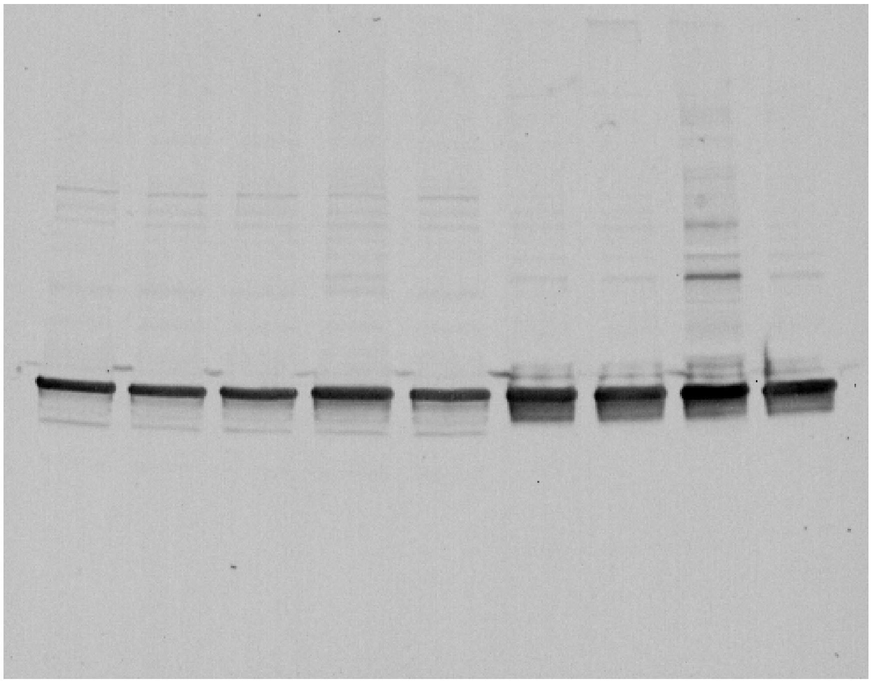

Supplementary Figure S3. Uncropped actin western blot image. A cropped portion of this actin western blot image is presented in Figure 3.

Supplementary Figure S4

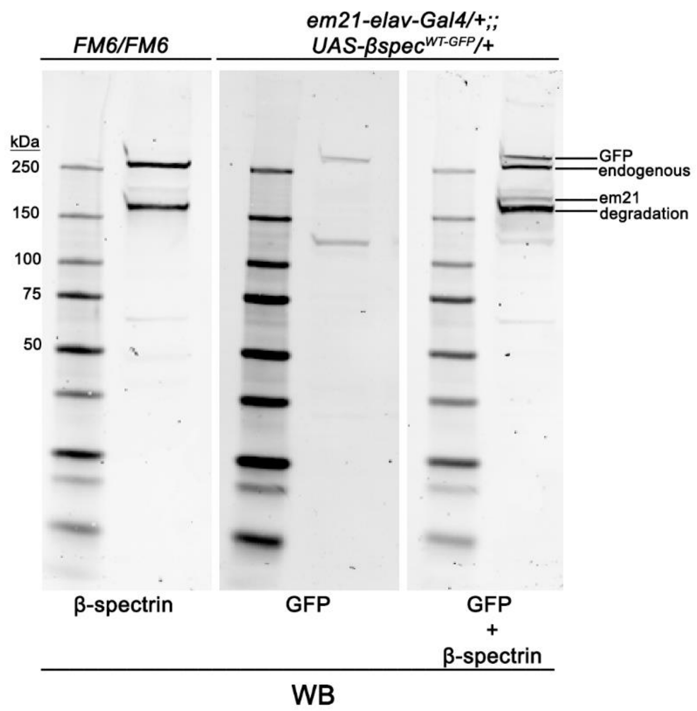

Supplementary Figure S4. β-spectrin-GFP fusion protein runs as a distinct, high molecular weight band relative to endogenous, untagged β-spectrin. Head extracts from *FM6/FM6* or *em21-elav-Gal4/+;;UAS-βspec*$^{WT\text{-}GFP}$/+ flies were subjected to SDS-PAGE followed by western blotting using GFP or β-spectrin antibody. *Left*, western blot using β-spectrin antibody of *FM6/FM6* flies expressing endogenous β-spectrin. Full-length endogenous β-spectrin runs near the 250 kDa protein ladder band, in agreement with the predicted molecular weight of 266 kDa. *Middle*, western blot using GFP antibody of *em21-elav-Gal4/+;;UAS-βspec*$^{WT\text{-}GFP}$/+ flies shows full-length β-spectrin-GFP runs above the 250 kDa protein ladder band, in agreement with the larger, 293 kDa size of the fusion protein. *Right*, re-probing of the GFP blot (middle panel) with β-spectrin antibody reveals the lower molecular weight endogenous β-spectrin protein. Thus SDS-PAGE resolves β-spectrin-GFP and endogenous β-spectrin proteins as two distinct bands.

Supplementary Figure S5

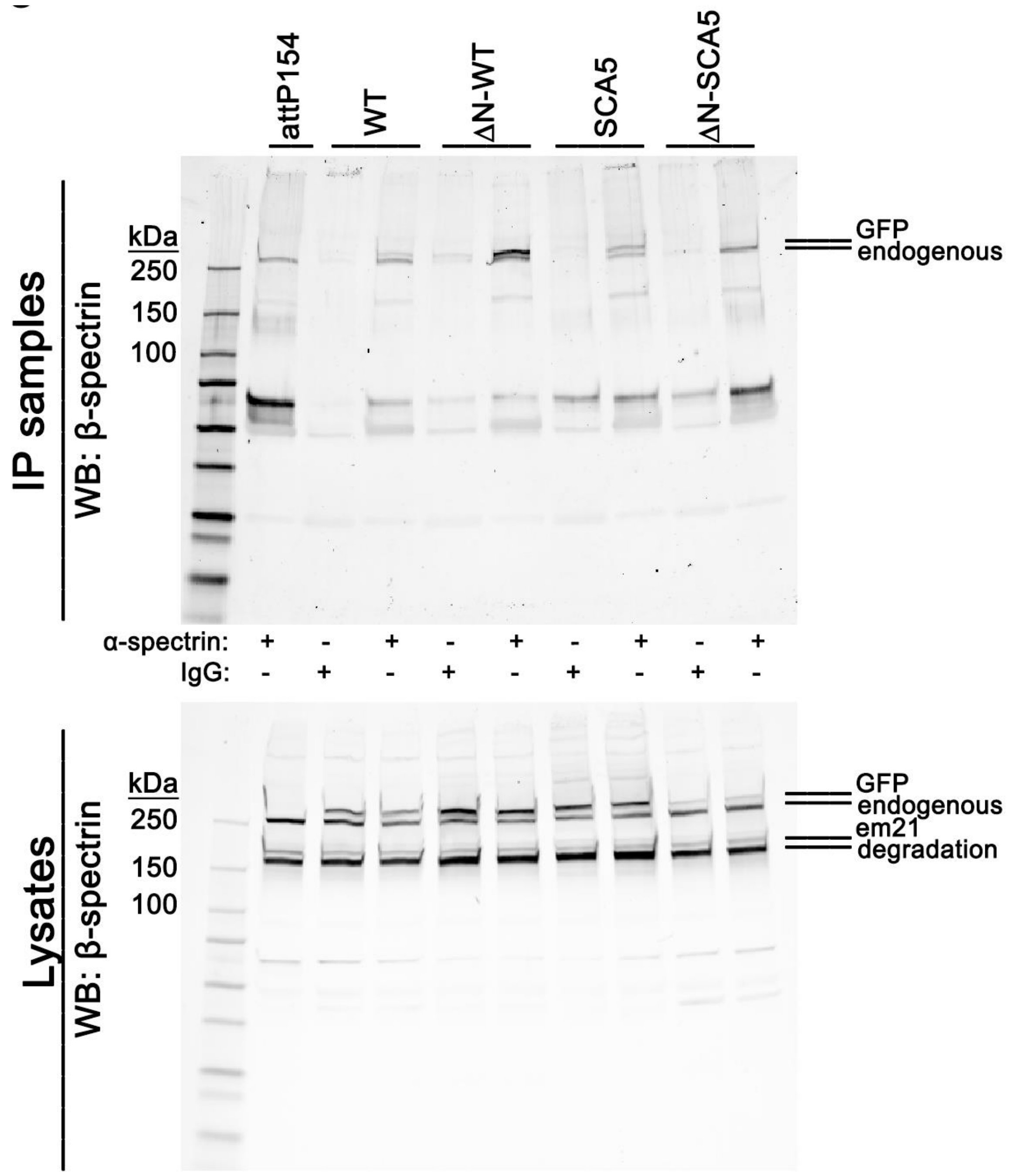

Supplementary Figure S5. SCA5 and N-terminally truncated β-spectrin mutants bind α-spectrin. Head lysates were prepared from *em21-elav-Gal4/+;;attP154/+* control flies or *em21-elav-Gal4/+;;UAS-βspec*^{GFP}*/+* flies expressing different β-spectrin-GFP fusion proteins. Immunoprecipitaiton was performed using α-spectrin or control IgG antibodies. In co-IP samples, endogenous β-spectrin (~250 kDa) is enriched in α-spectrin antibody samples relative to control IgG samples. Higher molecular weight wild-type, SCA5 and ΔN-β-spectrin-GFP

fusion proteins are likewise enriched in the α-spectrin antibody co-IP samples relative to IgG control. ΔN-SCA5 β-spectrin-GFP, which is present at low abundance in lysate, is present as a faint band in the α-spectrin antibody co-IP sample, but not in controls (IgG or attP154).

Supplemental Figure S6

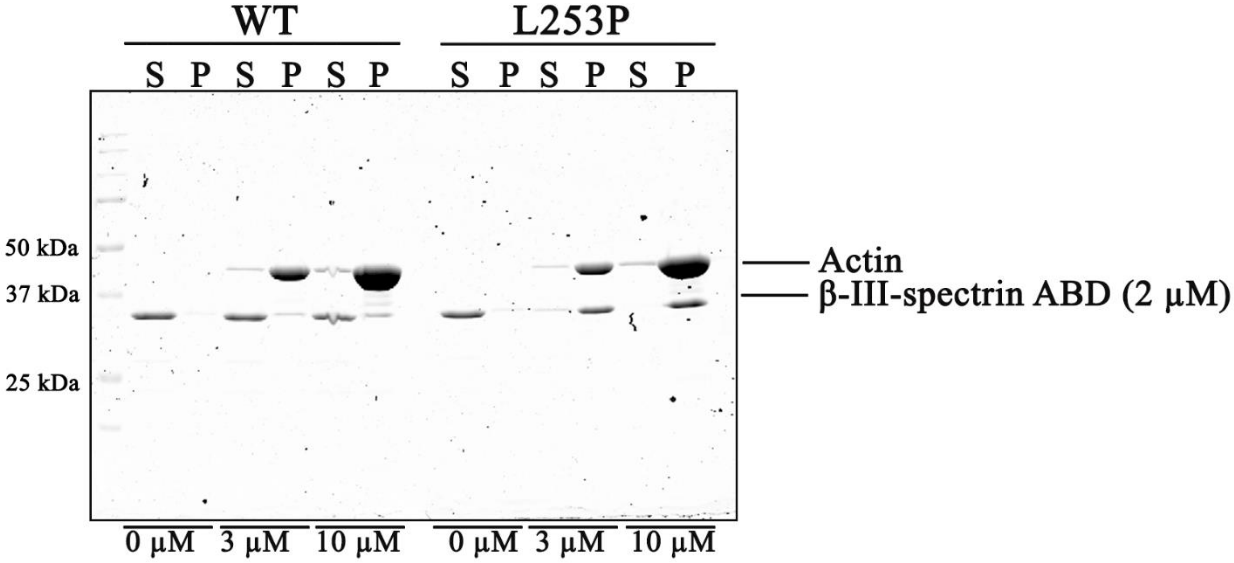

Supplemental Figure S6. Sedimentation of L253P ABD is dependent on F-actin. Actin co-sedimentation reactions were performed for wild-type (WT) and L253P mutant ABD with varying amounts of F-actin. After ultracentrifugation to pellet F-actin, supernatant (S) and pellet (P) samples were collected, followed by SDS-PAGE and Coomassie blue staining. In the absence of F-actin, wild-type and L253P ABDs (2 μM) are present in the supernatant samples, and only a trace amount of either ABD is detected in the pellet samples. In reactions containing 3 or 10 μM F-actin, almost all L253P ABD is in the pellet, consistent with high-affinity binding. In contrast, in reactions containing 3 or 10 μM F-actin, only a small amount of wild-type ABD shifts to the pellet, consistent with low affinity binding. For all reactions containing F-actin, the the majority of F-actin is present in the pellet. The trace amount of actin in the supernatant may reflect the G-actin that is in equilibrium with F-actin.

Supplemental Figure S7

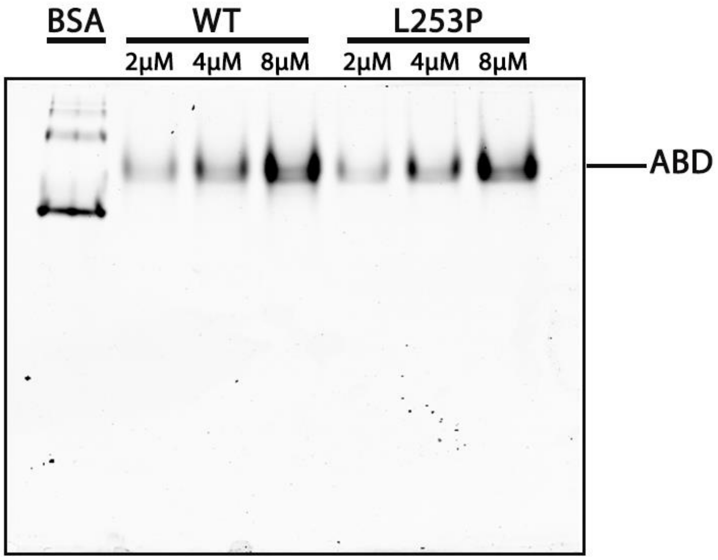

Supplemental Figure S7. Wild-type and L253P ABDs show similar band pattern on native gel. Wild-type or L253P ABD, at different concentrations (2, 4 or 8 μM), were loaded onto a 12% native polyacrylamide gel. Following electrophoresis, Coomassie blue staining was performed. The wild-type and L253P ABD proteins show similar band patterns. This suggests that the wild-type and L253P ABD proteins are in a similar monomeric/oligomeric state. In contrast, bovine serum albumin (BSA) separates into distinct bands, as previously described[3].

References

1. Neisch, A. L., Pengo, T., Avery, A.W., Li, M-G., Hays, T.S. Dynein acts to cluster glutamate receptors and traffic the PIP5 kinase, Skittles, to regulate postsynaptic membrane organization at the neuromuscular junction. *bioRxiv* doi:10.1101/2021.09.27.462070 (2021).
2. Dubreuil, R. R., Wang, P., Dahl, S., Lee, J. & Goldstein, L. S. Drosophila beta-spectrin functions independently of alpha-spectrin to polarize the Na,K ATPase in epithelial cells. *J Cell Biol* **149**, 647-656, doi:10.1083/jcb.149.3.647 (2000).
3. Kurien, B. T., Scofield, R. H. (eds.) Electrophoretic separation of proteins: methods and protocols. Methods in Molecular Biology. **1855**, 87-89, doi: 10.1007/978-1-4939-8793-1_8 (2019).
